# Postpartum depression crisis since the second lockdown and ‘screening paradox’: many women identified, very few treated

**DOI:** 10.1186/s12889-022-14705-5

**Published:** 2022-12-20

**Authors:** Magdalena Chrzan-Dętkoś, Tamara Walczak-Kozłowska

**Affiliations:** 1 Division of Developmental Psychology and Psychopathology, Institute of Psychology, Department of the Social Sciences, University of Gdańsk, Gdańsk, Poland; 2 Division of Neuropsychology, Institute of Psychology, Department of the Social Sciences, University of Gdańsk, Gdańsk, Poland

**Keywords:** Coronavirus, Postpartum depression, Perinatal mental health, Lockdown, Pandemic

## Abstract

**Objective:**

Exposure to stressful situations, such as emergencies, infectious diseases, and natural disasters, may lead to a heightened risk of perinatal mental health problems. Declared on March 11th, 2020, the global COVID-19 pandemic triggered an additional burden on women in the perinatal period. Safety recommendations, such as social distancing and isolation, were opposite to the usual advice given to new mothers. Besides fear, changes in financial stability and daily life reorganization contributed to increased depressive symptoms. As the periods of epidemic waves and lockdowns were associated with a more significant burden for young families, we aimed to assess the intensification of depressive and anxiety symptoms during the pandemic concerning the time intervals of the three lockdowns introduced in Poland. Methods: 1588 postpartum women took part in the online self-assessment with the Edinburgh Postnatal Depression Scale (EPDS) and General Anxiety Disorder 2 (GAD-2) questionnaire between January 1, 2020, and March 31, 2021. This self-screening is a part of a prevention program The Next Stop: Mum, implemented in the North of Poland.

**Results:**

The highest severity of PPD symptoms and anxiety were observed during the second lockdown in Poland: the mean score in the EPDS and anxiety assessment was significantly higher than the mean scores from previous pandemic periods. Since the second lockdown, the average EPDS and GAD-2 scores remained similarly high. Moreover, with the duration of the COVID-19 pandemic, the percentage of women with elevated symptoms of postpartum depression and anxiety began to increase. However, the Polish National Health Fund data indicate that only 0,7% of women giving birth in the northern macro-region of Poland received diagnosis and help from public funds. In The Next Stop: Mum project, 250 women benefited from psychological consultations.

**Conclusion:**

Increased severity of depression and anxiety symptoms during the pandemic indicates the need for additional psychological support for postpartum women. However, very few women are diagnosed in health facilities in the first year postpartum and thus are rarely referred for further treatment. The study shows that the availability of services and the focus on social and individual barriers may be critical factors in implementing perinatal mental health programs and practices. This may be especially needed in a country where the screening obligation is new. In case of a further pandemic, policymakers and health care professionals should be aware that the duration of the restrictions and the repetition of lockdowns are associated with the aggravation of symptoms. The online screening without the possibility to discuss the results is only partially effective in increasing referrals for possibly affected women.

## Introduction

In 2020, the World Health Organization declared a public health emergency of the new CoronaVirus Disease 19 (COVID-19). The rapid spread of the virus and the negative consequences of the infection on physical and mental health, which were gradually revealed with more and more research results, were the reasons why people began to live in constant anxiety for their health and the health of loved ones. This epidemic and its consequences have profoundly impacted the people who are particularly concerned about health - women in the perinatal period [8, 10, 11, 21, 27, 29]. In addition to the anxiety and stress directly associated with the fear of coronavirus infection and restrictions [26] parents in the perinatal period faced a double challenge: typical reorganization of life with a new baby and profound changes in everyday life with introduced restrictions which could provoke social isolation, lack of support and increased anxiety. The limitations of the appointments to only urgent emergency visits or replacing direct consultations with short teleconsultations, suspension of planned family labour, uncertainty in which hospital the delivery will take place and often the impossibility to deliver the baby in the planned hospital because of quarantine introduced among hospital staff, replacement of maternity units with units for patients with covid − 19 etc. introduced anxiety and stress [4, 25, 30]. Systematic reviews and meta-analyses conducted since the beginning of the pandemic reported an overall prevalence of depression and anxiety symptoms among women in the perinatal period ranging from 17 to 31% and 26.6–42%, respectively [8, 10, 11, 27, 29]. In addition, the prevalence of co-morbid symptoms of depression and anxiety was estimated to be about 18–20% [11, 29]. In general, the perinatal period is recognized as a time of heightened vulnerability with 1 in 5 women developing a mental health problem [14, 33]. Postpartum depression (PPD) and anxiety are leading diagnoses. PPD has long-term adverse effects not only on women but also on their family system as well as on the whole society – via high treatment costs [2]. Pandemic restrictions in health facilities mentioned above deprived many mothers of mental health screening, early diagnosis, and support. The evidence from different time points (e.g. [4, 8, 10, 11, 13, 18, 19, 27, 29]) pointed to the increased prevalence and severity of the symptoms of postpartum mental health disorders since the outbreak of the COVID-19 pandemic. However, we did not find any study that would track the severity of PPD and anxiety symptoms among women seeking postpartum support with regard to the lockdown periods, and it seems that such research is particularly needed [20]. It could allow policymakers and health care professionals to provide an evidence-based foundation for understanding the impact of lockdowns during the COVID-19 pandemic and possible future pandemics on women’s postpartum mental health.

Although many countries have a long tradition of screening for depression in the perinatal period, Poland introduced an obligation to screen for PPD in January 2019. On its basis, the Polish Ministry of Health implemented the project: The Next Stop: MUM co-financed by the European Social Fund, no. POWR 05.01.00-00-0023/18-00 aimed at screening for postpartum mental health disorders and offering early psychological intervention. In addition to screening procedures implemented at hospitals and primary health facilities, implementers of the The Next Stop: Mum project built an online platform for women seeking mental health support in the postpartum period. With this online formula, women can independently and anonymously self-assess the severity of postpartum depression symptoms and receive immediate feedback. If a high result is obtained, a woman is allowed to take advantage of the free-of-cost psychological consultations near the place of her residence or online. Implementing the program with the idea of quick psychological appointments is an important element in preventing postpartum mental health problems. The standard waiting period for a visit to a psychiatrist or psychotherapist covered by the National Health Fund in Poland ranges from three weeks to eight months in large cities from the three provinces covered by the The Next Stop: Mum program. During the COVID-19 pandemic, the waiting time was even longer. The situation is even more challenging in rural areas with minimal access to professionals. The situation of the pandemic and introducing the possibility of online psychological consultations, paradoxically, could make it easier for mothers in need to use this form of help. However, from the data from many European countries, we know that effective perinatal mental health support is still challenging even in countries with a long tradition of mental health screening. Webb et al. [32] study pointed out several barriers to implementing effective perinatal mental health policy, such as stigma, cultural beliefs, but also individual factors such as the absence of family support surrounding mental health, a paucity of awareness or knowledge about perinatal mental health, beliefs about medication, a reluctance or inability to attend mental health services or additional personal difficulties. According to Cox et al. [6] only 30.8% of women with PPD are identified in clinical settings, 15.8% receive treatment, and 6.3% receive adequate treatment. The The Next Stop: Mum project’s offer seemed to have met the needs of postpartum women: remote screening for PPD and the possibility of participating in free-of-cost, immediate remote psychological consultations.

The data from the online platform of The Next Stop: Mum project, were analyzed to trace the intensity of postpartum depressive symptoms and anxiety concerning the time intervals of the three lockdowns introduced in Poland. We also analyzed the frequency of attending psychological consultations and the symptoms in a group of mothers attending psychological consultations.

## Materials and methods

This online cross-sectional study aimed to identify potential changes during the COVID-19 pandemic in the severity of depressive and anxiety symptoms among postpartum women (up to a year after delivery). Information about the online assessment and psychological consultations were spread through hospitals, health clinics, posters, leaflets, internet advertisements, and midwives during home visits in the north macroregion of Poland. Participants were asked about sex, date of birth, and age. Other information such as level of education, perceived socioeconomic status, biomedical information etc. was optional. However, from further analysis, data from women who did not provide information about the level of education, place of residence and perceived socioeconomic status of the family were excluded.

The Ethics Board approved this study protocol for Research Projects at the University of Gdańsk (decision no. 20/2019). The ethics committee indicated that all adult patients had been deemed ethically and medically capable of consenting to participate in the research presented in this manuscript. The participants were informed that they also consented to participate in the study by completing the electronic version of the EPDS questionnaire. The informed consent was obtained from every participant of the study. All methods were carried out per the Code of Ethics of the World Medical Association (Declaration of Helsinki) for experiments involving human data collection.

We also collected data on the number of postpartum psychological consultations during the COVID-19 pandemic. Additionally, we asked the National Health Fund to provide data on the number of diagnoses of depressive disorder made to women by psychiatrists in the first year after childbirth in the three provinces covered by the program. Unfortunately, we could only obtain data for the Warmian-Masurian and Pomeranian province; for the Kuyavian Pomeranian, we were refused such information.

### Participants

The The Next Stop: Mum project covers 37 primary healthcare centers and seven state hospitals. During covid pandemic, when home and primary healthcare centres visits were limited, the midwives and nurses advised new mothers to fill an online questionnaire. Additionally, the project website was promoted on social and traditional media. Initially, we gained data from 3356 people who made self-assessments with the EPDS. From these data, we had to exclude assessments made by men, those women who gave birth over a year after childbirth, and those participants who were not inhabitants of the three provinces covered in our program. We also excluded from further analysis data from women who did not provide information about the level of education, place of residence and perceived socioeconomic status of the family. Eventually, we analyzed data from 1747 women:

- *n* = 161, between January 1, 2020, and March 15, 2020,
- *n* = 134, between March 16, 2020, and May 24, 2020,
- *n* = 247, between May 25, 2020, and October 22, 2020,
- *n* = 340, between October 23, 2020, and January 15, 2021,
- *n* = 650, between January 16, 2021, and March 19, 2021,
- *n* = 215, between March 20, 2021, and March 31, 2021.

All women were up to a year after giving birth, capable of reading and writing in Polish, with a mean age of 30.45 years (*SD* = 4.52, Min. = 16, Max. = 45). Most women (77%) had higher education, 19% - secondary, 2% - vocational and 2% - primary. 47% of participants were residents of big cities (over 250,000 inhabitants), 21% lived in medium-sized cities (between 50 and 250,000 inhabitants), 14% lived in small cities (up to 50,000), and 18% lived in the countryside.

Out of these 1747 women participating in the assessment with EPDS, 1588 also filled the GAD 2 assessment.

### Method

#### Edinburgh postnatal depression scale

The Edinburgh Postnatal Depression Scale (EPDS) by Cox et al. [5] was used to detect the severity of PPD symptoms in women. This short (10-item), self-reporting tool is recommended as a screening method for the needs of medical personnel taking care of women during the perinatal period. Points in the EPDS range from 0 to 30 and are commonly interpreted regarding two cut-off points:

- 10–11 points: slightly increased severity of PPD symptoms,
- 12 or more points: increased severity of PPD symptoms (requiring extended clinical assessment).

#### Generalized anxiety disorder 2-item

The Generalized Anxiety Disorder 2-item (GAD-2) is a brief and easy-to-perform initial screening tool for generalized anxiety disorder [16]. It consists of two questions, and respondents answer on a four-point Likert scale. Points in GAD-2 range from 0 to 6. A score of 3 points is the preferred cut-off for identifying possible cases and in which further diagnostic evaluation for generalized anxiety disorder is warranted. Using a cut-off of 3, the GAD-2 has a sensitivity of 86% and specificity of 83% for the diagnosis of generalized anxiety disorder.

#### Desk research

We also analyzed the existing data on the number of psychological consultations provided for postpartum women in the *blinded* program during the COVID-19 pandemic.

Additionally, we used data obtained from the National Health Fund on the occurrence of diagnoses coded F.53 (Postpartum depression) or F.32 (Depressive episode) in ICD-10 received by women in the first year postpartum (data was from the Warmian-Masurian province) in 2018 (before COVID-19 pandemic and before the implementation of the *blinded* program) and from 2020 to 2021 (COVID-19 pandemic and the program under implementation).

### Statistical analysis

We used SPSS Version 26.0 for statistical analyses. Intergroup differences were tested with the one-way ANOVA and post hoc Tukey’s tests. The occurrence of clinical results was described concerning the cut-off points [5].

## Results

### The severity of PPD symptoms during the pandemic lockdowns

**Fig. 1:**
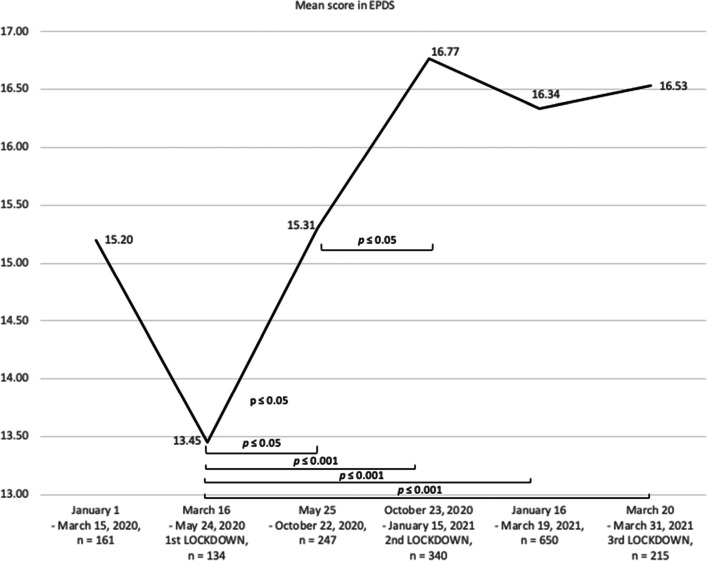
Mean scores obtained by Polish women during on-line self-assessment with the EPDS

One-way ANOVA revealed that scores obtained by women in the EPDS self-assessment differed significantly in different COVID-19 pandemic periods (*F* = 8.03, *p* < .001). The results of the post hoc analyses are presented in Fig. 1.

The highest severity of PPD symptoms among self-screened postpartum women in The Next Stop: Mum project was observed during the second lockdown in Poland (October 23, 2020 – January 15, 2021). The mean score in the EPDS was significantly higher in comparison to the mean scores from previous periods of the pandemic. Since the second lockdown, the average EPDS score has not changed significantly i.e. remained at a similarly high level. The lowest severity of PPD symptoms was observed during the first lockdown in Poland (March 16 - May 24, 2020). However, the average result in EPDS did not differ significantly from the average result obtained in the period preceding the pandemic (January 1 - March 15, 2020) but differed from the subsequent period (“easing of restrictions”: May 25 - October 22) - thus possibly indicating a gradual worsening of PPD symptoms.

In addition, we were able to observe that with the duration of the COVID-19 pandemic, the percentage of women with elevated symptoms of postpartum depression began to increase (see Table 1), reaching approx. 80% during the second lockdown and remaining without significant changes in subsequent time points of the assessment.

**Table 1:** Occurrence of the clinical scores regarding the cut-off points in the online self-assessments with the EPDS

Time period of the COVID-19 pandemic	Scores obtained in the EPDS assessments		
	Normal range (0–9 points)	Slightly increased (10–11 points)	Increased (12–30 points)
January 1 - March 15, 2020, *n* = 161	16.1%	13.0%	70.8%
March 16 - May 24, 2020, 1st LOCKDOWN, *n* = 134	32.8%	11.9%	55.2%
May 25 - October 22, 2020, *n* = 247	16.2%	12.6%	71.3%
October 23, 2020 - January 15, 2021, 2nd LOCKDOWN, *n* = 340	11.8%	7.9%	80.3%
January 16 - March 19, 2021, n = 650	12.6%	8.8%	78.6%
March 20 - March 31, 2021, 3rd LOCKDOWN, *n* = 215	12.6%	8.4%	79.1%

### The severity of generalized anxiety during the pandemic lockdowns

**Fig. 2:**
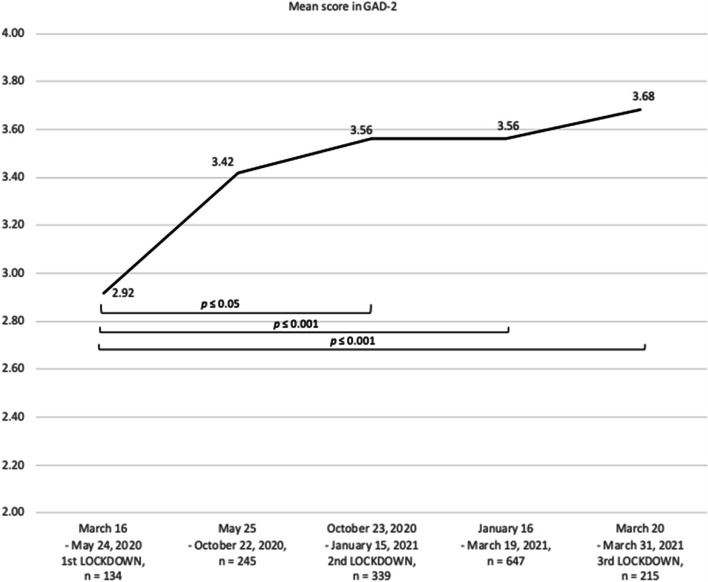
Mean scores obtained by Polish women during online self-assessment with the GAD-2

One-way ANOVA revealed that scores obtained by women in the GAD-2 self-assessment differed significantly in different COVID-19 pandemic periods (*F* = 4.81, *p* < .001). The results of the post hoc analyses are presented in Fig. 2 (the pre-pandemic period is not included in the figure because the question was not added to the questionnaire until the 1st lockdown).

The severity of GAD-2 increased since the first lockdown in Poland (March 16, 2020 – May 24, 2021): the mean scores in the GAD-2 obtained by women since the 2nd lockdown differed significantly from the mean score obtained in the first lockdown. Since the second lockdown, the average GAD-2 score has not changed significantly i.e. remained at a similarly high level.

64.4% of women reached the cut-off point (3 points and above) in GAD-2. The detailed analysis is described in Table 2.

**Table 2:** Occurrence of the clinical scores, regarding the cut-off points, in the online self-assessments with the GAD-2

Time period of the COVID-19 pandemic^a^	Scores obtained in the GAD-2 assessments	
	Normal range (0–2 points)	Increased (3–6 points)
January 1 - March 15, 2020, *n* = -	N/A	N/A
March 16 - May 24, 2020, 1st LOCKDOWN, *n* = 134	50.0%	50.0%
May 25 - October 22, 2020, *n* = 245	62.0%	38.0%
October 23, 2020 - January 15, 2021, 2nd LOCKDOWN, *n* = 339	64.9%	35.1%
January 16 - March 19, 2021, *n* = 679	67.1%	32.9%
March 20 - March 31, 2021, 3rd LOCKDOWN, *n* = 215	66.5%	33.5%

^a^The pre-pandemic period is not included in the table because the question was not added to the questionnaire until the 1st lockdown

### Psychological consultations and the occurrence of postpartum depression diagnoses in one out of three provinces covered by the <blinked> program

In Tables 3 and 4 data obtained from the regional National Health Fund are presented. For Warmian – Masurian province (Table 3) we obtained data separately for F.53 (Postpartum depression) and F.32 (Depressive episode), for Pomeranian province we obtained these data together (Table 4).

**Table 3:** The occurrence of depression diagnoses among postpartum women in the Warmian-Masurian province - one out of three provinces of Poland covered by the < The Next Stop: Mum > program

Number of patients with ICD10 diagnosis	Year			
	2018	2019	2020	2021 (up to June 30)
F.53 (Postpartum depression)	7	7	6	2
F.32 (episode of depression)	30	69	90	72

The population of the Warmian-Masurian province is approx. 1,4 million people (data from December 31, 2020). On average, around 13,000 children are born in the Warmian-Masurian province each year (data from December 31, 2020)

**Table 4:** The occurrence of depression diagnoses among postpartum women in Pomeranian province - one out of three provinces of Poland covered by the < The Next Stop: Mum > program

Number of patients with ICD10 diagnosis	Year			
	2018	2019	2020	2021 (up to June 30)
F.53 + F. 32	163	169	166	53

The population of the Pomeranian province is approx. 2,324 million people (data from December 31, 2020). On average, around 24,495 children are born each year (data from December 31, 2020)

Since the outbreak of the COVID-19 pandemic until March 31, 2021, we have provided 434 psychological consultations for 250 postpartum women. Approx. 90% of the consultations were provided remotely.

## Discussion

This study investigated the severity and the prevalence of depression symptoms and anxiety in women in the first year postpartum willing to perform remote self-screening for PPD and anxiety during the COVID-19 pandemic. Our aim was to track the severity and the prevalence of the PPD and anxiety symptoms with special regard to the lockdown periods in Poland, as it has not been investigated yet.

The highest severity of PPD and anxiety symptoms was observed during the second lockdown in Poland. Additionally, since the second lockdown, the average EPDS score remained at a similarly high level with a mean score reaching 16.34–16.77 points, and almost 80% of self-screened women obtained results above the clinical cut-off point. Similarly, in the case of anxiety symptoms, since the second lockdown, the average GAD-2 score had not changed significantly (equalled 3.56–3.68), with about 33–35% of self-screened women obtaining results above the clinical cut-off point. These results correspond with other research outcomes: for instance, studies conducted in Canada, China, Turkey, and Italy report that the rates of depression and anxiety in pregnant women are more than doubled compared to studies before the pandemic [9, 15, 17, 34]. In the general population, more than 42% of people surveyed by the US Census Bureau in December 2020 reported symptoms of anxiety or depression, and it is a significant increase compared to 11% in the previous year [1]. In Poland, Gambin et al. [12] showed that the highest depression and general anxiety severity was experienced in May and December 2020. However, their study did not contain data from the 3rd lockdown.

Although the difference between the mean EPDS score obtained by postpartum women during the first lockdown and the mean score obtained during the period preceding the pandemic was not statistically significant, some decline could have been observed in the PPD severity trajectory chart. Perhaps this initial decline reflects the country’s first ‘positive’ response to the pandemic: the perceived benefits of the transition to remote work, such as a partner working remotely could provide additional support for a woman staying with her newborn baby, the authorities’ reassurance that the world will be able to deal effectively and quickly with the coronavirus pandemic through isolation. Further increase of the PPD symptoms and anxiety may reflect a growing fear of an increasingly spreading and unstoppable pandemic, as well as mental overload associated with the pandemic restrictions.

Initially, the The Next Stop: Mum program was oriented to help midwives in their new vocational responsibility: conducting screening for postpartum mental health problems, yet it turned out to be particularly helpful during the COVID-19 pandemic. There is, however, an important observation to note: though many women performed self-screening on our online platform, it is worrisome that only 250 of them benefited from the psychological consultations offered in the project. In Poland, we obtain even lower rates of initiating treatment than those mentioned by Cox et al. [6]: less than 1% of women in postpartum period is referred and starts treatment.

It is difficult to verify whether it is due to the COVID-19 pandemic or to the implementation of new depression screening procedures, which are now required as an essential part of Poland’s new perinatal care standard. the number of women diagnosed with depression in the first year after childbirth is frighteningly small: statistically, about 15–20% of women suffer from depression [14, 33]. Given the potentially serious consequences of untreated anxiety and depression during the perinatal period on mothers and their offspring [28], the implementation of evidence-based interventions should be addressed.

Our study’s elevated rate of clinical results in EPDS and GAD 2 assessment heightens the importance of support and treatment for women in the perinatal period during the pandemic and can be a base for elaborating mental health prevention program in case of future pandemics.

Although psychological interventions for preventing and treating depression and anxiety during the perinatal period are effective [3, 7], the problem may still be the awareness of the problem. Several actions can be recommended to help reach potentially affected women. First, research is warranted to develop tailored and evidence-based eHealth interventions during the perinatal period to ensure broad access [24]. Secondly, the healthcare providers, but also policymakers should be aware of the problem and the possible solutions. For instance, in their article Motrico et al. [21] provided recommendations for perinatal mental health providers during the COVID-19 pandemic, which include screening, promotion of self-help strategies and possibility of self-referral to the local psychological centers. However, the analysis of attendance to psychological consultations offered in the Next Stop: Mum program and the data from the National Health Fund shows that it is rather not the availability of the specialists but the referral itself is a major problem. The review of the literature revealed two main themes that could explain this situation. The first is associated with the specificity of thinking in depression. In Moritz et al. study [22] current level of depression and well-being predicted attitudes toward treatment, suggesting that when the patient feels more depressed, doubts about the effectiveness of therapy emerge more strongly, which influences the decision to start treatment. Even in countries with a longer history of screening such as United Kingdom, in April 2018, 24% of women still had no access to specialists in perinatal mental health services. Once a woman was offered treatment, a barrier to accessing this treatment was a reluctance or inability to attend treatment due to a lack of time, childcare, and transport. Other factors that affected access to treatment included additional personal difficulties and little family support [32]. A German study by Tomczyk [31] suggests another explanation: among people who knew where to find a psychologist or psychotherapist, anticipated self-stigma emerged as a significant barrier to seeking help. As mentioned earlier [32], self-stigma, especially in the postpartum period, may be associated with a fear of being labelled (or self-labelled) as “a bad mother’ or ‘a mother who can’t cope’, and thus can be the main factor associated with a low rate of referral and seeking support. Additionally, early motherhood, when the identity of oneself as a mother is developing, is a sensitive time concerning the mother’s self-esteem. Asking for support, and help, and acknowledging that the new reality may be different than expected can be fearsome, associated with a fears of not being ‘a good enough’ and result in denial of the difficulties. Based on this short review of the literature we suggest that campaigns addressing the stigma and universality of the problem could be helpful to overcome these barriers.

The Covid-19 pandemic was a specific period when women obtained information about their mental state mostly through the online platform. A personal conversation about the results was not possible, and that could influence the rate of referral. According to Webb et al. [32] a coalition of health visitors, midwives, general practitioners, family doctors, or primary care physicians, improving access to psychological therapies, practitioners, psychologists, and psychiatrists is needed to encourage referral and reduce the risk of women falling through care pathway gaps. The possibility of creating such a multidisciplinary team could be helpful in reaching vulnerable women.

### Limitations

Due to the observational nature of this study, the data should not be considered as derived from the general population of postpartum women, as these were women willing to screen for PPD during the COVID-19 pandemic. The specificity of this group is evidenced by the generally high results obtained in the EPDS self-assessment by the participants in this study. It would have been difficult to obtain a representative sample of the screening assessments conducted in direct contact as these were often omitted in many facilities during the pandemic due to the need for medical staff to focus on priority tasks. However, attention should be paid to the value of the results obtained in this study in the context of the general trend indicating that the severity of symptoms increases with the duration of the COVID-19 pandemic, and thus screening assessments may be even more needed than before the pandemic.

## Conclusions

The severity and prevalence of depressive and anxiety symptoms increase with the duration of the COVID-19 pandemic, reaching a critical point in the second lockdown and maintaining its high intensity in the following months, which can reflect the greater burden experienced by women along with the duration of the pandemic.

The rates of depression symptoms observed in remote online self-screening compared to the number of consultations provided in the prevention program and data obtained from the National Health Fund on the prevalence of depression diagnoses in first year postpartum is alarming. Although many women achieve significantly elevated screening results, very few women start treatment in health facilities: even when it is free and available both online and close to home. This shows the importance of psychological factors which are active in the decision about the referral: such as lack of hope associated with the depressive mood, self-stigma, and shame.

### Recommendations

1. The duration of the pandemic was associated with the increase of depressive and anxiety symptoms in mothers in the first year postpartum. In case of a further pandemic, policymakers and health care professionals should be aware that the duration of the restrictions and the repetition of lockdowns are associated with the aggravation of symptoms.
2. The medical staff should be aware of the low number of referrals in the group of women who screened positive for PPD.
3. Not only the availability of services, but focus on social and individual barriers may be critical factors in implementing perinatal mental health programs and practices. This may be especially needed in a country where the screening obligation is new.
4. The online screening without the possibility to discuss the results is only partially effective to increase referrals for possibly affected women. he possibility of conversation about the score obtained in screening and efficacy of treatment and self-care as well as maternal doubts regarding referral to psychologists or psychiatrists can be an important step in encouraging women to seek and start treatment.

## Data Availability

The datasets used and/or analysed during the current study are available from the corresponding author on reasonable request.
